# A systematic review of strategies to increase access to health services among children in low and middle income countries

**DOI:** 10.1186/s12913-017-2180-9

**Published:** 2017-04-05

**Authors:** Tess Bright, Lambert Felix, Hannah Kuper, Sarah Polack

**Affiliations:** International Centre for Evidence in Disability, London School of Hygiene & Tropical Medicine, London, UK

**Keywords:** Access, Health care, Children, Low and middle income country, Universal health coverage

## Abstract

**Background:**

Universal Health Coverage is widely endorsed as the pivotal goal in global health, however substantial barriers to accessing health services for children in low and middle-income countries (LMIC) exist. Failure to access healthcare is an important contributor to child mortality in these settings. Barriers to access have been widely studied, however effective interventions to overcome barriers and increase access to services for children are less well documented.

**Methods:**

We conducted a systematic review of effectiveness of interventions aimed at increasing access to health services for children aged 5 years and below in LMIC. Four databases (EMBASE, Global Health, MEDLINE, and PSYCINFO) were searched in January 2016. Studies were included if they evaluated interventions that aimed to increase: health care utilisation; immunisation uptake; and compliance with medication or referral. Randomised controlled trials and non-randomised controlled study designs were included in the review. A narrative approach was used to synthesise results.

**Results:**

Fifty seven studies were included in the review. Approximately half of studies (49%) were conducted in sub-Saharan Africa. Most studies were randomised controlled trials (*n* = 44; 77%) with the remaining studies employing non-randomised designs. Very few studies were judged as high quality. Studies evaluated a diverse range of interventions and various outcomes. Supply side interventions included: delivery of services at or closer to home and service level improvements (eg. integration of services). Demand side interventions included: educational programmes, text messages, and financial or other incentives. Interventions that delivered services at or closer to home and text messages were in general associated with a significant improvement in relevant outcomes. A consistent pattern was not noted for the remaining studies.

**Conclusions:**

This review fills a gap in the literature by providing evidence of the range and effectiveness of interventions that can be used to increase access for children aged ≤5 years in LMIC. It highlights some intervention areas that seem to show encouraging trends including text message reminders and delivery of services at or close to home. However, given the methodological limitations found in existing studies, the results of this review must be interpreted with caution.

**Systematic review registration:**

PROSPERO CRD420160334200

**Electronic supplementary material:**

The online version of this article (doi:10.1186/s12913-017-2180-9) contains supplementary material, which is available to authorized users.

## Background

### The problem, condition, or issue

The substantial gap between the need for health care and the level of access in Low and Middle Income Countries (LMIC) is well established. In 2015, the World Health Organisation (WHO) estimated that 5.9 million deaths occurred in children under 5 years of age, with a disproportionate concentration of deaths occurring in LMICs [1]. More than half of child deaths worldwide can be prevented through access to simple and affordable interventions [2–4]. However, coverage and access to these interventions remains low in LMIC, particularly among the poorest groups [3].

Since the 1978 Alma Ata declaration expressed the need for action to ensure “Health for All” by the year 2000, many resolutions and goals have been endorsed with the ultimate objective of achieving what is now known as Universal Health Coverage (UHC) [5–9]. UHC is defined as “ensuring that all people have access to needed promotive, preventive, curative and rehabilitative health services, of sufficient quality to be effective, while also ensuring that people do not suffer financial hardship when paying for these services” [10]. Despite progress made towards achieving UHC and remarkable health gains, evidence suggests that many children in LMIC are still not accessing needed health care services [3, 6].

The benefits of increasing health coverage go beyond prevention of deaths in children. Delayed or lack of access to health services for children can lead to a worsening of health, which, in turn, can negatively impact their ability to attend school, social interaction and quality of life [11]. Further, the economic impact on the caregiver can be substantial, through lost days of work, and higher long term health care costs, ultimately contributing to poverty [12].

There are many contributing factors to the underutilisation of effective health interventions in LMIC [13]. Children may experience particular barriers as they are often dependent on a parent or caregiver to access services. Barriers may arise on the demand side, through individual, household or community level factors, or the supply side, through health systems characteristics [14, 15]. According to the widely recognised conceptual framework by Peters et al. (2008) healthcare access can be considered as involving the following four dimensions, each with a supply and demand component, and these need to be considered when devising strategies to overcome barriers to care [12, 14]:

1. **Geographical accessibility:** relates to the physical distance and/or travel time from the health service to the user. If services are concentrated in particular areas and inadequate provision is available in others (e.g. in poor, rural areas) this imposes a geographic barrier [15].
2. **Availability of health care:** relates to the ability to access the right care at the right time. This element includes factors such as the hours of operation of a service, the availability of specialist staff, and waiting times that meet the user’s demand for services.
3. **Financial accessibility**: refers to affordability to access a service that depends on costs and prices of services, and user’s resources and willingness to pay. This also includes the indirect costs such as opportunity costs of time of both the patient and those accompanying them.
4. **Acceptability**: is dependent on the characteristics and structure of health services matching the needs and expectations of the users as well as individual user’s knowledge and attitudes.

The dimensions of Peters’ framework are not mutually exclusive and may interact with each other. Thus, strategies to improve health care access can either be simple, targeting just one dimension (e.g. improving local availability of health services) or complex, incorporating multiple interacting components [16]. For example, geographical accessibility can be improved by better transportation, which would then depend on financial accessibility, i.e., the ability of users to pay for the transport. Furthermore, Jacobs’ et al. (2012) extended Peters’ framework highlighting that interventions to increase health care access can target supply or demand side and can be financial or non-monetary [14].

While many studies have explored and identified a range of barriers to accessing health care, the evidence for the effectiveness of appropriate interventions to overcome these barriers and increase access to health care for children is unclear [13]. A limited number of previous reviews have focussed on specific intervention types (e.g. cash transfers [17], and pay for performance [18]), however these reviews did not specifically focus on children in LMIC. Identifying strategies that aim to increase health care access for children and understanding their effectiveness is key for informing policy and the implementation of appropriate evidenced based interventions for this group [17–21].

We conducted a systematic review of interventions to increase access to health services among children aged ≤5 years LMIC. The specific objectives were to:

- Identify and describe the different strategies used to increase access to health care services
- Evaluate the effectiveness of the strategies used to increase access to health care services

The systematic review was performed according to the Preferred Reporting Items for Systematic Reviews and Meta-Analysis (PRISMA) statement [22].

## Methods

### Procotol and registration

The study protocol is registered with PROSPERO International prospective register of systematic reviews (registration number: CRD420160334200).

### Study eligibility criteria

Studies with the following characteristics were included in the review:

#### Types of participants

Studies were included if children aged ≤5 years were the main recipients of the intervention or if the intervention was directed at carers and/or health professionals (e.g. text message reminders) but the outcome (e.g. child immunization) was aimed at children. We focussed on children 5 years and under because they form a distinct group with unique health needs.

#### Types of interventions

According to the Peters’ and Jacobs’ frameworks, we included supply- and demand- side interventions that sought to increase access to health care for children. Access to health care was defined for the purposes of this review as the receipt of health care among people who could potentially benefit from it and included health promotion, disease prevention, diagnosis, care for episodic and chronic illness, and rehabilitation services [4]. Packages of interventions were included as long as at least one component aimed to increase health care access for children. Interventions promoting breastfeeding were not included.

#### Types of outcome measures

We included studies that reported on at least one of the following outcome measures for children:

- **Health care utilisation:** e.g. proportion of children taken to health facility in event of illness, uptake of early infant diagnosis of Human Immunodeficiency Virus (HIV)
- **Immunisation uptake:** e.g. coverage of Diphtheria, Pertussis, and Tetanus (DPT) vaccination, measles vaccination
- **Compliance with medication/referrals** e.g. intermittent preventative treatment for malaria, adherence to antiretroviral therapy (ART)

#### Types of study

We included randomised controlled trials (RCTs) for which the unit of randomisation was cluster or individual, and non-randomised controlled study (NRS) including non-randomised control trials (non-RCT), controlled before and after studies, quasi RCTs, historically controlled studies and interrupted-time-series studies. Study designs were defined using the Cochrane Handbook [23].

### Information sources

Four databases (EMBASE, Global Health, MEDLINE, and PSYCINFO) were searched in January 2016. The search strategy comprised five concepts: population; intervention-settings; intervention-strategies; study design; and country. Search terms were developed using MeSH (see Appendix 1). The search was limited to include all literature up to December 2015. No limits were placed on language. Reference lists of included studies were inspected in order to further identify relevant studies. Furthermore, studies included in any relevant systematic reviews were reviewed for relevance. Finally, if any study protocols were identified, a search was made to determine whether the results of the study had been published.

### Search

The strategy used for the EMBASE database is shown in Appendix 1. This strategy was applied across all databases; however, it was adapted to fit the relevant subject headings for the particular database.

### Study selection

All studies identified through the search process were exported to a bibliographic database (EndNote version X7) for removal of duplications and screening. Three review authors (TB, SP, and HK) independently examined the titles, abstracts, and keywords of electronic records according to the eligibility criteria. One author examined all titles and abstracts (TB), whilst the remaining records were divided between two authors (SP, HK) for double screening. Results of the initial screening were compared and full-text records obtained for all potentially relevant studies. Two review authors (TB and SP) screened the full texts using eligibility criteria for final inclusion in the systematic review. Any disagreements in the selection of the full text for inclusion were resolved by discussion with a third author (HK).

### Data extraction and analysis

Data were extracted into a Microsoft Excel database developed for the purposes of this review. The first author (TB) extracted all data and this was independently checked by a second author (SP).

Data were extracted on the following study components:

1. Publication details: author, year and journal.
2. Methods: study design and duration.
3. Study location: including country and setting (urban/rural).
4. Participants: age, sex and sample size.
5. Interventions: details on the intervention and its comparator.
6. Outcomes: type of outcome(s), measurement instruments, and time points measured.
7. Results: including relevant measure of effect (odds ratio, risk ratio, p values).
8. Targeted barrier: the interventions were classified according to the barriers to access that they addressed using the Peters’ conceptual framework.

In classifying the effectiveness of the interventions, study results were classified as “positive” if there was a statistically significant improvement in the outcome(s) of interest (health care utilisation, immunisation uptake and/or compliance outcomes) in the intervention group relative to the comparison group. Studies which found a statistically significant decrease in the outcome(s) relative to the comparison group were classified as “negative”. If there was no statistically significant change in the outcome of interest, studies were classified as “null”. Finally, studies measuring multiple outcomes were classified as “mixed positive” if there was a significant improvement in at least one outcome and no significant change in other outcomes and “mixed negative” if findings were a mix of negative and null.

A narrative approach was used to synthesise results in line with the recommendations for systematic reviews of complex interventions [24]. We did not conduct a meta-analysis due to the variation in included study designs, intervention types and outcomes.

### Risk of bias in individual studies

Two authors (TB, SP) independently assessed the methodological quality of the selected studies. Disagreements were resolved through discussion. For randomised control trials, we used the Cochrane ‘Risk of Bias’ tool [25]. For non-randomised studies, we used the Effective Public Health Practice Project (EPHPP) quality assessment tool for quantitative studies [26].

## Results

### Study selection

**Fig. 1:**
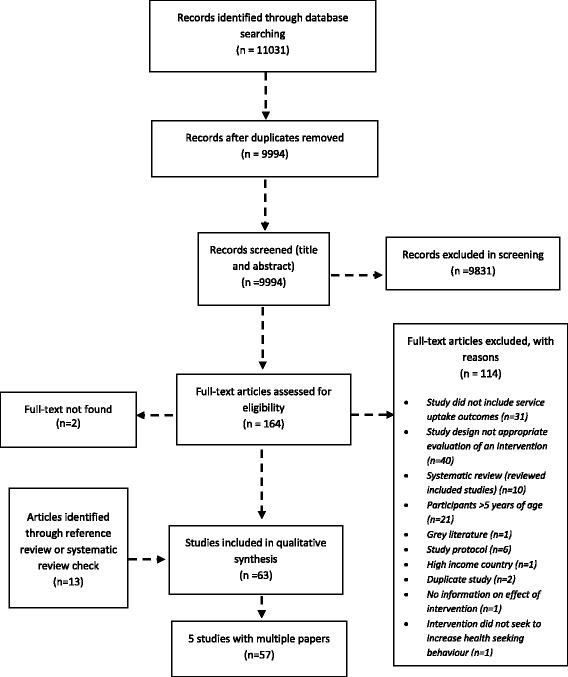
Flow chart of search results

A total of 11,031 records were initially identified by the electronic searches, of which 1037 were duplicates and removed. A further 9,882 records were excluded during the initial screening yielding 164 potentially eligible studies for which full text reports were sought. Following the full text review, 114 studies were excluded and the full text could not be located for 2 articles [27, 28]. An additional 13 studies were identified through screening reference lists of the included publications, yielding a total of 63 publications for inclusion in the review. Five of these were duplicate publications on the same study and these were grouped together leaving a total of 57 included studies. Figure 1 shows the PRISMA flow chart.

### Study characteristics

**Fig. 2:**
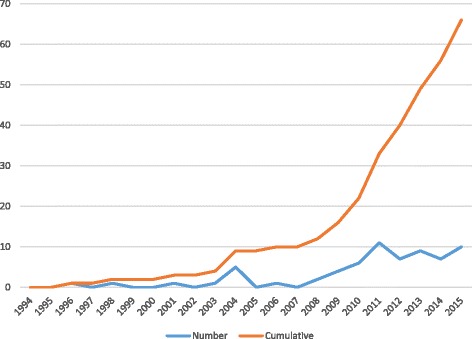
Year of publication of included studies

A summary of the study characteristics of the included studies is shown in Table 1. Studies were published between 1996 and 2015 (Fig. 2). The majority of studies were published from 2010 onwards (*n* = 40; 70%). Study duration ranged from 3 months to 6 years.

**Table 1:** Characteristics of included studies

Variable	Number	Percent
Location		
Urban or periurban	14	25
Rural or semi rural	34	59
Mixed	9	16
Decade of publication		
1990	2	4
2000	15	26
2010	40	70
Study design		
RCT	44	77
Non-RCT	2	4
Controlled before-after study	8	14
Historical controlled study	2	4
Interrupted time series	1	2
Region		
Latin America/Caribbean	4	7
East Asia/Pacific	3	5
Sub-Saharan Africa	28	49
South Asia	21	37
Middle East/North Africa	1	2
Outcome category		
Immunisation	20	35
Health care utilisation	27	47
Compliance	2	4
Combination	8	14
Intervention category		
Delivery of services closer to or at home	7	12
Health promotion/education programme	23	40
Service level improvements	10	18
Text messages	5	9
Financial or other incentives	12	21

Approximately half of the studies (*n* = 28, 49%) were conducted in Sub-Saharan Africa, 21 (37%) in South Asia, four (7%) in Latin America/Caribbean, three (5%) in East Asia/Pacific, and one (2%) in the Middle East/North Africa. More than half of the studies (*n* = 34, 59%) were conducted in rural or semi-rural locations, 14 (25%) were carried out in urban or peri-urban centres and 9 (16%) were carried out in a combination of settings (eg. urban and rural).

The majority of studies (*n* = 44; 77%) were RCTs. The remaining studies employed the following NRS study designs; non-RCTs (*n* = 2; 4%); historical controlled study (*n* = 2; 4%); controlled before-after study (*n* = 8; 14%) and interrupted time series analysis (*n*= 1; 2%).

#### Participants

The sample size of 39 studies which reported outcomes for individual participants ranged from 180 to 12,326 children with a median of 1205 and combined total of 70,900. The remaining 18 studies reported on the number of households, villages, health centre or births/pregnancy outcomes.

#### Outcome types

Of the 57 included studies; 27 (47%) reported health care utilisation outcomes, 20 (35%) reported on immunisation, 2 (4%) studies reported compliance outcomes and 8 (14%) studies reported on multiple outcomes (e.g. health service utilisation and compliance). Additional file 1 highlights the specific outcomes measured for each study.

### Risk of bias within studies

A total of 44 included studies used a RCT design (either cluster RCT or RCT). The majority of studies (*n* = 40; 90%) were judged as having an unclear risk of bias in at least one of the six domains (sequence generation, allocation concealment, blinding of outcome assessment, incomplete outcome data, selective reporting and other bias). The remaining studies, 4 (10%) were judged as having a high risk of bias. The domains most commonly contributing to high bias risk were lack of blinding of outcome assessors, incomplete outcome data and ‘other’ biases such as recall bias and use of self-reported data to ascertain vaccination status or health care utilisation (see Appendix 2). Most of the 13 NRS studies were assessed to be of moderate quality (i.e. weak in one domain) (*n* = 8), and strong quality (*n* = 1), and the remaining 4 were judged as weak using the EPHPP tool. The main sources of bias in NRS studies were study design, presence of confounders, and withdrawals or dropouts (Appendix 2).

### Description of studies

#### Comparison group

The vast majority of studies (*n* = 44; 77%) compared interventions to standard care/usual services. Six studies (10%) applied a simplified version of the intervention to the control group [29–31]. One study (2%) compared two different treatment methods [32]. Three studies (6%) compared two different intervention delivery modes [33–36]. One study (2%) used historical controls [37]. In two studies, both intervention and control groups received part of an intervention (e.g. systems strengthening) and only the intervention group received the main component of the intervention (3%) [38, 39].

#### Type of interventions

The interventions identified in this review were grouped into broadly similar categories and into supply-side or demand-side and non-monetary and financial categories according to the Jacobs’ framework [14]:

- *Supply side: non-financial*
  - ○ **Delivery of services at or closer to home (** ***n*** **= 7**) [32, 33, 36, 40–43]: including delivery of immunisation, medication/treatment, and referrals by health care professionals, community health workers (CHW), and immunisation camps
  - ○ **Service level improvements (** ***n*** **= 8)** [30, 34, 44–50]: including health worker training, scaling up services, and integration of services
- *Supply side: financial*
  - ○ **Service level improvements (** ***n*** **= 2)** [51, 52]: including contracting in or out of services, and pay for performance
- *Demand side: non-financial*
  - ○ **Health promotion/education programmes (** ***n*** **= 23)** [11, 31, 38, 39, 53–72]**:** delivered by varying personnel including health workers, CHW, and participatory women’s groups
  - ○ **Text messages (** ***n*** **= 5)** [37, 73–76]: including text message reminders, and promotion of service
- *Demand side: financial*
  - ○ **Financial or other incentives (** ***n*** **= 12)** [77–90]**:** including cash transfers, vouchers, fee exemptions and food incentives

Some studies evaluated interventions with demand- and supply-side components (combined interventions) and these were allocated to one of the above categories according to their primary component for simplicity. With regards to Peters’ framework, 49% of studies used interventions that targeted more than one dimension of access (*n* = 28), with the remaining targeting a single dimension. A summary description of all the interventions in the included studies is provided in Tables 2 and 3.

**Table 2:** Description of interventions of included studies, grouped according to supply-side and non-financial and financial (P = positive; MP = mixed positive; N = negative; U = unclear)

	Non-financial	Effectiveness	Reference	Financial	Effectiveness	Reference
Supply	Delivery of services close to home			Service level improvements		
	*Home visits by nurse or other health worker*			Pay for performance for health care workers	MP	
	Home visits by nurse to provide immunisation to those who did not attend appointments	P	[41]	Contractor delivery of primary health services (contracting-out vs contracting-in)	U	[52]
	Home visits by weighing agent who flagged abnormalities with GP and those in need provided with free consultations	P	[42]			
	*CHW*					
	Diarrhoea (ORS)	P	[43]			
	Malaria (IPTc)	P				
		MP	[32]			
		N				
	*Immunisation camps*					
	Well publicised immunisation camps and food incentives	P				
	Service level improvements					
	*Health worker training*					
	Health worker training	P	[46]			
		MP	[30]			
	*Scaling up of services*					
	Strengthening of routine vaccination programme function	N	[47]			
	*Integration of services*					
	Integration of intermittent preventive treatment for children alongside EPI vaccines	P	[45]			
	Integration of HIV services with immunisation/ANC	P	[34]			
		N	[48, 50]			
	*Combined interventions (Primary component service level improvement)*					
	Health worker training, health systems improvements, family and community activities (eg. formation of village health workers)	MP	[44]			
	Integration of HIV and immunisation services, operational support, training for staff, counselling of caregivers, community awareness campaigns	N	[91]

**Table 3:** Description of interventions of included studies, grouped according to demand-side and non-financial and financial (P = positive; MP = mixed positive; N = negative)

	Non-financial	Effectiveness	Reference	Financial	Effectiveness	Reference
Demand	Health promotion/education programmes			Financial or other incentives		
	*Health worker*			*Cash transfers*		
	Redesigned immunisation card, centre based education delivered by health worker	P	[70, 71]	Cash transfers (conditional or unconditional)	MP	[78, 86]
	Structured educational programme on childhood infections for mothers delivered by health worker	P	[58]		N	[81, 82]
	Post-partum home visits by registered midwives to provide information, educate and support women	N	[11]	*Fee exemptions*		
	*CHW*			User fee exemption	P	[77]
	CHW home visits for pregnant women to promote newborn care, refer sick newborns	N	[38]		MP	[79]
		MP	[57]	*Incentive schemes*		
	Package of essential newborn care for pregnant women delivered by CHW	N		Food/medicine coupon incentive at each immunisation visit	P	[83]
	Postnatal educational programme delivered by CHW	N	[55]	Supplementary nutrition as monthly take home for children attending paediatric HIV/AIDS clinic	P	[85]
	Educational programme for mothers using pictorial cards about vaccinations delivered by CHW	P		*Combined interventions (primary component financial)*		
	Antenatal and postnatal home visits for pregnant women by CHWs to provide health messages	MP	[63, 68]	Fee exemption, social mobilisation, education, improvement of service quality, financial monitoring	P	
	Antenatal and postnatal home visits for pregnant women by CHWs to provide health messages, assist with birth in absence of skilled care, manage illness where referral not available (sepsis, pneumonia), health facility strengthening	N	[72]	Conditional cash transfer, strengthening of services	N	[84]
				Conditional cash vouchers, health service strengthening and community based nutrition programme	MP	[87]
	*Other member of the community (teacher, volunteer, lay counsellor)*					
	Educational programme on newborn care	N				
	Educational programme on vaccines	MP	[53]			
		N	[67]			
	*Women’s groups*					
	Women’s groups with participatory models of communication, identification of problems, development, implementation and monitoring of strategies to improve maternal and neonatal problems	N	[59, 61, 64, 66, 69]			
	*Combined interventions (Primary component education)*					
	Women’s groups, health systems strengthening, training of staff	N	[39]			
	Health promotion delivered by CHW, illness management, reporting, community development	P				
	Health education of families, identification of sick newborns in the community by CHW, health systems strengthening and strengthening of referral systems (including provision of free care and referrals)	P	[54]			
	Home visits by CHWs, training in improved case management of sick children, women’s groups, strengthening of health systems	N	[65]			
	Text messages					
	Early infant diagnosis	P	[37]			
	HIV appointment reminders	P	[74]			
	Vaccination	P				
		MP				
	Text messages providing health promotion for HIV	P				

#### Effectiveness of interventions

The effectiveness of the interventions is summarised by intervention type:

### Supply-side; non-financial

#### Delivery of services close to home

**Fig. 3:**
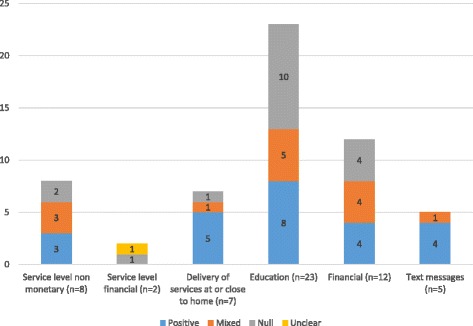
Summary results of included studies by intervention type

As shown in Table 2, the seven studies in this group included interventions delivered by health professionals [41, 42], CHW [32, 33, 35, 36, 43], and an immunisation camp [40]. In total five (71%) of the seven studies showed a statistically significant improvement in uptake in the outcome measures of interest (health care utilisation, immunisation and/or compliance outcomes), one (14%) had mixed-positive significance (i.e. significant improvement and no significant change on at least one outcome measure respectively) and one (14%) had no significant impact on any of the outcome measures of interest (null effect) (Fig. 3; Additional file 1). There were no clear trends in intervention effectiveness between the different delivery modes (e.g. health professional or CHWs).

#### Service level improvements

There were eight studies evaluating service level improvements which included health care worker training [30, 44, 46], scaling up of services [47] and integration of services such as HIV and immunisation services [34, 45, 48–50]. Overall, three of these studies were classified as positive (37.5%), three as mixed-positive (37.5%) and two as null 25%) (Fig. 3; Additional file 1). There was some variation in effectiveness between the specific intervention approaches: the two health worker training interventions showed a significant improvement in all (*n* = 1) or in at least one (*n* = 1) of the outcomes of interest (mixed-positive significance). Two of the three studies evaluating service integration were positive [34, 45], and one showed null effect. One study assessed scaling up services and found no significant impact on immunization coverage [47].

Two studies within this group were evaluated interventions with more than one component (combined interventions). In one such study, health worker training was conducted alongside health systems improvements, and family and community level activities and showed mixed-positive results [44]. The other study combined integration of HIV and immunisation services, operational support, caregiver counselling and community awareness campaigns and found null effect on health care utilisation and immunisation uptake [91].

### Supply-side; financial

#### Service level improvements

Two studies were identified that evaluated financial service level improvements (Table 2). A cluster RCT study of pay for performance for health care workers showed a significant improvement in health care utilisation and no significant impact on immunization coverage (mixed-positive) [51]. A non-randomised trial evaluated delivery of primary health services by a contractor but the results were unclear [52].

### Demand-side; non-financial

#### Health promotion/education programmes

Health education/promotion programmes were the most frequently evaluated intervention identified in this review (*n* = 23) (Table 3). Four educational interventions were delivered by health professionals (nurses or doctors) and addressed immunization and childhood infections [11, 58, 70, 71]. In 11 studies, education of families and caregivers by CHW was provided on a variety of topics including: newborn care, antenatal care and vaccinations [29, 31, 38, 54–57, 62, 63, 65, 68, 72]. Women’s participatory learning groups that aimed to identify maternal and neonatal problems and strategies to improve these were evaluated in five studies [39, 59, 61, 64, 66, 69]. In the remaining three studies, educational programmes on topics including vaccinations and newborn care were delivered by another member of the community (e.g. teacher or peer) [53, 60, 67].

Overall, 8 of the 23 (35%) educational interventions were associated with a positive effect, five (22%) were mixed-positive and ten (43%) showed null effect (Fig. 3). Three of the health worker delivered educational interventions had either a positive or mixed-positive impact and one showed null effect. Results of studies where education was delivered by CHWs, teachers, peers, or counsellors were more varied (Table 3; Additional file 1). None of the five studies evaluating women’s groups as a standalone intervention found a significant improvement in our outcomes of interest. Further, one study combined women’s groups with health systems strengthening and staff training and found no effect [39].

Of the seven studies that evaluated health education delivered by a CHW, the majority found null effect (*n* = 4; 57%), one (14%) was positive, and two (26%) were mixed-positive. A further three studies combined health education delivered by a CHW with other components. Positive results were seen when CHW education was combined with either: health systems strengthening, or community development [54, 92]. However, when CHW education was combined with both strengthening of health systems and women’s groups, no effect was seen [65].

#### Text messages

Five studies evaluated text messages reminders or promotion of a health care service (Table 3). Four of these studies evaluated text reminders for attending services for early infant diagnosis of HIV [37]; HIV care [74]; and vaccination [73, 76]. Of these studies, three were positive and one was mixed-positive (Fig. 3) [76]. Another intervention, which provided HIV related health promotion via text, was also positive [75].

### Demand-side; financial

#### Financial or other incentives

Financial interventions were the second most common intervention category identified in this review (*n* = 12) (Table 3). A range of interventions were tested: unconditional or conditional cash transfers [78, 82, 84, 86, 87, 90, 92], fee exemptions [77, 79, 80, 88, 89], and food incentive schemes [83, 85].

One third of studies in this group were positive, one third found mixed-positive results, and a third showed null effect (Fig. 3). Specifically, studies evaluating food incentive schemes all found a positive impact (*n* = 2). Studies evaluating removal of user fees alone were either positive (*n* = 1) or mixed positive (*n* = 1). In addition, fee exemptions in combination with social mobilisation, education, and strengthening of services, a positive impact was found on health care utilisation [93].

Results from cash transfer interventions (4 null and 2 mixed) were more varied (Table 3; Additional file 1). When conditional cash transfers were combined with health services strengthening, null effect was seen [84]. When combined with health services strengthening and a community based nutrition programme, mixed positive results were seen [87].

### Combined interventions

A total of nine studies were considered to evaluate interventions with multiple components. These studies combined demand and supply side interventions for example health education together with health systems strengthening. Although they included different interventions, most of these studies had a primary component which we used to assign to the relevant intervention groups in this review (see Tables 2 and 3). Overall, 44% of combined interventions had a primary component of health education, one third had a financial or other incentive component (*n* = 3), and 22% combined service level strengthening with other components (*n* = 2). Of the two combined interventions classified as supply-side which combined service level improvements with community components such as awareness campaigns, one found null effect on the outcomes of interest and the other had mixed positive results [44, 91]. Considering the seven combined interventions classified on the demand side, 43% (*n* = 3) were positive [54, 92, 93], 14% were mixed positive (*n* = 1) [87], and the remaining 43% found null effect (*n* = 3) [39, 65, 84].

**Fig. 4:**
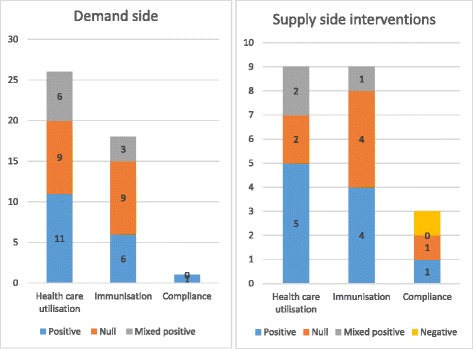
Summary results of included studies by outcome type and supply-side and demand-side interventions

The effectiveness of the interventions is also summarised by outcome type:

- **Health care utilisation:** Of studies evaluating the impact of supply side interventions on health care utilisation, 56% had a positive effect (null 22%; and mixed-positive 22%). For the demand side interventions, 42% of the studies were positive, (null: 35%; and mixed-positive results 23%) (Fig. 4).
- **Immunisation uptake:** Of studies evaluating immunisation uptake, 44% of studies targeting the supply side were positive, 44% showed no effect, and the remaining 11% found mixed-positive significance. On the demand side, results were more varied; 33% found evidence of improved immunisation uptake, 50% found null effect, and 17% found mixed-positive significance.
- **Compliance outcomes:** Of studies measuring compliance outcomes, 33% of studies targeting supply-side factors were positive, 33% found mixed-positive significance, and 33% found negative results. The study with interventions targeting the demand side was positive (100%).

These results are summarised in Fig. 4. Note that some studies measured more than one of these outcomes, and hence the denominator for the percentages is based on the number of outcomes rather than study.

## Discussion

To the best of our knowledge, this is the first comprehensive systematic review of interventions to increase access to health care with a specific focus on children in LMIC. The review was large, comprising 63 peer-reviewed articles from 57 studies. The review identified the following six broad groups of interventions that aim to increase access to health services for children in LMIC, both on the demand-side and supply-side:

- Supply side; non-financial
  - ○ Delivery of services close to home
  - ○ Service level improvements
- Supply side; financial
  - ○ Service level improvements
- Demand side; non-financial
  - ○ Health promotion/education
  - ○ Text message reminders
- Demand side; financial
  - ○ Financial or other incentives

The interventions identified in this review target different dimensions of health care access, as characterised by the Peters’ and Jacobs’ frameworks, both on the supply and demand side. On the supply side, delivery of services at or closer to home (by nurses, CHWs, school programmes or camps) target both geographical barriers and financial barriers by reducing the travel and opportunity costs associated with attending health services. Interventions designed to improve health services tackle issues of acceptability and availability aiming to increase quality services that meet the needs and expectations of users.

On the demand side, the most common interventions identified in this review were health promotion/educational programmes via different delivery modes addressing acceptability (i.e. aiming to influence user’s knowledge and attitudes), as well as geographical accessibility barriers (i.e. providing health promotion within the home or community). Text message reminders or health promotion target the acceptability dimension of access through the improving user’s knowledge and attitudes about the service. Finally, a group of interventions target the financial accessibility of services through providing financial assistance, for example cash transfers, vouchers and fee exception, or food incentives conditional on certain health seeking behaviours.

Evidence on the effectiveness of the interventions included in this review were mixed, even within the different intervention types. The two intervention types most consistently associated with a positive improvement in the uptake of health services for children were the use of text messages (demand side; non-financial) and the delivery of services closer to home (supply-side; non-financial).

### Supply-side; non-financial interventions

#### Delivery of services close to home

In many LMIC settings, health centres are concentrated in urban areas making it logistically difficult and prohibitively expensive to reach for many people particularly in rural areas [3]. This review suggested that interventions aimed at addressing these geographical and financial accessibility barriers by bringing services closer to the home may be beneficial in terms of improving health care utilisation, immunisation and medication/referral compliance for children. For example, the use of CHW for delivery of services has been identified as one strategy to address the growing shortage of health workers in LMIC [93, 94]. CHW programmes are likely to improve cost-effectiveness of healthcare systems by reaching large numbers of previously underserved people with basic services at low cost. The term “community health worker” encompasses a range of community health assistants who are trained to work within the communities from which they come [93, 94]. Given that they are members of the community in which they work, this is thought to increase acceptability of services. Their role may involve provision of preventive or curative treatment or health education programmes. Further primary studies are needed to explore the long term sustainability and cost-effectiveness of these interventions. In addition, while all but one of the studies evaluating interventions that delivered services at or closer to home were RCTs, the quality was generally poor or unclear and therefore some caution in the interpretation is warranted.

#### Service level improvements

Non-monetary service level improvement interventions identified in this review included health worker training, integration of services, and scale up of services. Four studies that evaluated integration of services were identified, thought to target availability and acceptability of services. Findings were mixed, aligning with previous work by Briggs et al. (2006) [20]. Health worker training interventions included in this review were designed to target multiple dimensions of access, primarily availability, but also acceptability through community mobilisation. Overall, these interventions were associated with an increase in health care utilisation and immunisation uptake. This aligns with findings from a review by Willey et al. (2013) that found that health worker training had a positive impact on quality of care and coverage of services [95].

### Supply-side; financial interventions

#### Service level improvements

There were very few studies identified in this review that were classified as supply side financial interventions. Those that were identified used pay for performance, tackling availability and acceptability barriers, and contracting, which potentially targets all four barriers of access depending on available resources [14]. Health systems strengthening is seen as a global health priority and is particularly relevant for achieving UHC. Thus, further evidence is warranted on the effectiveness of these interventions that tackle inefficiencies within the health system and their impact on access for children.

### Demand-side; non-financial interventions

#### Health promotion/education

Our review found that educational interventions delivered by health care workers generally had a positive impact on health care utilisation or immunization uptake. We did not find evidence of significant improvements in health access outcomes for children associated with participatory women’s learning groups. This aligns to some extent with a meta-analysis of trials of women’s groups by Prost et al. (2013) which found a non-significant reduction in maternal and neonatal mortality across all the included trials [96]. However, in a sub-group analysis of 4 studies in which at least 30% of pregnant women participated in the groups found that the reduction in neonatal mortality was significant. Although such sub-group analyses were beyond the scope of this current review, this warrants further attention in the context of the impact of participatory women’s groups on access to health care for children. Evidence from this review suggested that educational programmes delivered by CHWs was varied. Previous reviews have suggested that this method is associated with improved immunisation uptake, and reduce childhood diarrhoea. [97, 98] The mixed impact on health care access as a result of educational programmes may reflect the broad range of delivery modes and intervention content as well as the variable quality of the studies. In addition, as health education is thought to principally target acceptability barriers, the impact of health promotion on access to health care may be limited if financial and geographical barriers prevent access to health services [14].

#### Text messages

With evolving mobile phone technology and rapidly increasing numbers of mobile phone users in LMIC, there is increased interest in the use of this relatively low cost technology within health services [99]. The generally positive impact of text messages to remind carers about appointments or send health promotional messages in studies included in this review concurs with previous systematic reviews which found mobile phone reminders generally improved attendance at health appointments among adults [100], health care outcomes (all ages) [99], and ART adherence among adults [101]. While it is encouraging that the majority studies of text messages in the current review were RCTs, the study quality was of some concern, echoing previous reviews [99, 100]. Further, all the studies were conducted in Sub-Saharan Africa, thus their generalisability to other settings is unclear. The use of text messages is potentially promising area for improving health care access for children in LMIC which deserves further attention. However, there is a need for more evidence in different geographical settings using well designed RCTs.

### Demand-side; financial interventions

#### Financial or other incentives

Financial or other incentives appear to tackle financial accessibility as participants do not incur fees for service or receive food at the health appointment. Conditional cash transfers may in addition tackle geographic accessibility by making money available for transport as well as acceptability through tackling issues of cultural preferences and stigma [14]. Our findings contrast somewhat to a systematic review by Lagarde et al. (2007) of conditional cash transfers for improving uptake of health services in LMIC, which concluded that these programmes are effective in increasing the use of preventive services [17]. However, the authors confirmed the dearth of evidence on the topic and many of the studies included were from grey literature sources or used study designs that did not meet our inclusion criteria. In addition, the previous review did not have a specific focus on children. Although there were few studies, the removal of user fees was associated with a positive or mixed positive outcome. As discussed by Jacobs’ et al. (2012), user fee removal could result in reduced access if increased drug supply is not considered [14].

### Combined interventions

We delineated the studies identified in the review into the categories above to allow clarity about which interventions could be used to tackle different dimensions of access. However, it is important to recognise that barriers to accessing health care are rarely constrained to the demand or supply side alone. It is likely that both demand and supply side barriers, as well multiple dimensions of access, need to be addressed simultaneously through a ‘package’ of interventions in order to maximise the chances of a positive impact on access to health services [14]. Despite this, we identified only nine studies which included interventions with multiple components. Among the combined interventions, our review did not find a consistency positive impact on access. However, there is also evidence to suggest that the effectiveness of intervention combinations depends substantially on the context. [14] For instance, our review identified two similar interventions that combined educational interventions delivered by CHW with health systems strengthening. The first study, conducted in Bangladesh, found a positive impact on care seeking for newborn illness, whilst the second, conducted in India, found no effect on this outcome or on the uptake of BCG vaccine [54, 65]. The differences in findings may be due to contextual factors that affect the interventions mechanisms, or the way in which the intervention was implemented in the different settings.

The lack of sufficient data on combined interventions may reflect the challenges faced in evaluating them. Further research into the effectiveness of complex interventions that include multiple components is required using guidance from bodies such as the Medical Research Council (MRC) [102].

### Recommendations

Although the quality of evidence was generally mixed, the review does highlight two intervention areas, in particular, that seem to show encouraging trends and which deserve further attention: text message reminders and delivery of services at or close to home.

This review has highlighted a need for further high quality research into the effectiveness of all intervention types identified in this review. These studies must be well-designed, conducted in a range of LMIC, and should consider context specific barriers the intervention aims to address. In particular, research into combined interventions, should be prioritised. As many of the interventions identified in the review can be considered as complex, involving several interacting components and targeting multiple dimensions of access, the use of MRC guidelines for evaluations of complex interventions may be beneficial [16]. For example, including process evaluations can help to understand the mechanisms of impact, both positive and negative, how the intervention was implemented, and contextual factors that shape aspects of the intervention. This, in turn, may help policy-makers evaluate how evidence from a different context could be applied in their setting.

### Strengths and limitations

We adopted a systematic approach to searching, screening, appraising and extracting data checked by two reviewers. We attempted to minimise citation bias through reviewing references of included studies and relevant systematic reviews.

There were some limitations that should be taken into account when interpreting the findings of this review. Although we did not restrict our search in terms of language, we only used English search terms and few French or Spanish citations were retrieved. Therefore, relevant evidence from francophone Africa and Latin America may have been missed. While the broad nature of our review question was effective in highlighting the range of different intervention approaches it precluded a detailed analysis of each intervention type and potential mechanisms to be theorised and this deserves further attention.

We included only peer-reviewed studies that employed RCT, non-RCT, controlled before after study, historically controlled study and interrupted time series designs to reduce risk of important biases. However, interventions addressing health care access are often complex and challenging to evaluate using a trial design. For instance, provision of an essential health package which has occurred in many low and middle income countries has not been evaluated in this way. We may therefore have missed interventions of interest evaluated using other study designs or published in grey literature.

This review did not explore the quality of the interventions that were delivered, or the impact on equity and thus warrants further investigation. Finally, the vast majority of studies included in this review did not assess cost-effectiveness of the interventions being studied. Further attention is needed to understand this aspect of these interventions.

## Conclusions

This review fills a gap in the literature by identifying the range and effectiveness of interventions that can be used to increase health care access for children in LMIC. It highlights some intervention areas that seem to show encouraging trends including text message reminders and delivery of services at or close to home. 

## Figures and Tables

**Fig. 5:**
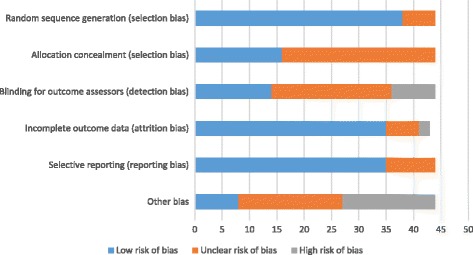
Summary of risk of bias in included RCTs

## Appendix 1: Search strategy

**Table 4:** EMBASE search strategy

Concept	Number of hits
A. Population	
1. child/	1529952
2. infant/	588992
3. exp paediatrics/	95214
4. (child* or infant* or p?ediatric*).ti,ab	1934384
5. exp handicapped child/	8130
6. (“children with disabilit*” or “people with disabilit*” or pwd or “persons with disabilit*” or “individuals with disabilit”).ab,ti.	6255
7. exp adolescent/	1328092
8. “adolescen*”.ti, ab.	258384
9. 1 or 2 or 3 or 4 or 5 or 6 or 7 or 8	3306882
B. Intervention - Setting	
10. exp health program/	98631
11. exp health service/	4080400
12. exp health promotion/	76003
13. exp rehabilitation/	291352
14. exp immunization/	257019
15. exp health care/	3838165
16. (“health adj5 access” or “community hospital” or “health care” or “health services” or “rehabilitat*” or therap* or treatment).ab,ti.	6644190
17. 10 or 11 or 12 or 13 or 14 or 15 or 16	9451205
C. Intervention - Strategies	
18. (barrier* or facilitator* or uptake or usage or intake or access* or adherence or compliance or complian* or adher* or promot* or increas* OR prevent* or reduc* or program* or educat* or campaign* or predict* or determin* or behavio#r*).ab,ti.	12511153
19. ((barrier* or facilitator* or uptake or usage or intake or access* or adherence or compliance or complian* or adher* or promot* or increas* OR prevent* or reduc* or program* or educat* or campaign* or predict* or determin* or behavio#r*) adj3 (health* or ill or illness or ills or well or wellbeing or wellness or poorly or unwell or sick* or disease*)).ab,ti.	576682
20. 18 or 19	1251153
D. Study design	
21. Clinical trial/	859727
22. exp controlled clinical trial/	530729
23. exp experimental design/	12337
24. exp experiment/	2254758
25. exp feasibility study/	61100
26. “clinical trial”.ab,ti.	129856
27. “controlled clinical trial”.ab,ti.	12879
28. “randomi#ed controlled trial”.ab,ti.	70586
29. randomi#ed.ab,ti.	588698
30. (trial or rct).ab,ti.	582657
31. “intervention study”.ab,ti.	8364
32. “quasi randomi#ed”.ab,ti.	3127
33. ((clin* or control* or compar* or evaluat* or prospectiv*) adj3 (trial* or studi* or study)).ab,ti.	2116839
34. 21 or 22 or 23 or 24 or 25 or 26 or 27 or 28 or 29 or 30 or 31 or 32 or 33	5055412
E. Country	
35. exp Developing Country/	83201
36. (asia or africa or “south america” or “developing count*” or “low middle income countr*”).ti.	55505
37. 35 or 36	125953
A + B + C + D + E	
38. 9 and 17 and 20 and 34 and 37	1961

## Appendix 2: Risk of bias of included studies

**Table 5:** Summary of risk of bias of included studies for non randomised trials, controlled before after studies, interrupted time series, and historically controlled trials (1=strong, 2=moderate, 3=weak, -=Not applicable)

Study author, Year	Selection bias	Design	Confounders	Blinding	Data collection methods	Withdrawals/ dropouts	Global rating
Brenner et al, (2011) [92]	1	1	3	2	1	-	2
Chandir et al, (2010) [83]	2	2	1	2	-	3	2
Fatugase et al, (2013) [58]	2	2	1	3	2	1	2
Finocchario-Kessler et al, (2014) [37]	1	3	1	2	-	1	2
Galasso et al, [35]	2	3	3	2	1	3	3
Kundu et al, (2012) [85]	1	3	3	2	1	1	3
McCollum et al, (2012) [34]	2	2	3	2	-	1	2
Oche et al, (2011) [67]	1	2	3	2	-	1	2
Ridde et al, (2013) [93]	2	2	3	2	-	3	2
Robinson et al, (2001) [46]	3	2	3	2	-	-	3
Ryman et al, (2011) [47]	2	2	3	2	-	-	2
Schwartz et al, (2004) [52]	2	2	3	2	-	-	3
Simonyanm et al, (2013) [42]	1	2	1	2	-	1	1

Note: Data collection methods “not applicable” when validity and reliability were not of tools were not a concern (e.g. self-report or medical records). Withdrawals/dropouts “not applicable” when surveys were conducted on a different group of people at baseline and follow-up

## Additional file

Additional file 1: Summary of results of included studies. (DOCX 127 kb)

## Abbreviations

Adj: Adjusted
ANC: Antenatal care
AIDS: Autoimmune Deficiency Syndrome
ARV: Antiretroviral
BCG: Bacillus Calmette–Guérin (TB vaccine)
C: Compliance
CHW: Community Health Worker
CI: Confidence Interval
CBA: Controlled Before After study
DBS: Dry Blood Spot
DPT/DPT1/DPT3: Diphtheria Pertussis and Tetanus
EPI: Expanded Programme on Immunisation
GP: General Practitioner
HCU: Health Care Utilisation
Hep B: Hepatitis B
HIV: Human Immunodeficiency Virus
I: Immunisation
IPT: Intermittent Preventive Treatment
IPTc: Intermittent Preventive Treatment (children)
IPTi: Intermittent Preventive Treatment (infants)
LMIC: Low and Middle Income Country
MMR: Measles Mumps Rubella
NGO: Non-Government Organisation
NS: Not Significant
PCR: Polymerase Chain Reaction
PCV13: Pneumococcal Conjugate Vaccine
PMTCT: Prevention of Maternal to Child Transmission
OR: Odds Ratio
ORS: Oral Rehydration Solution
OLS: Ordinary Least Squares
(c)RCT: (Cluster) Randomised Control Trial
RCH: Reproductive and Child Health
RDT: Rapid Diagnostic Test
RR: Risk Ratio
SE: Standard Error
SMS: Short Message Service
TBA: Traditional Birth Attendant
UHC: Universal Health Coverage
VHW: Village Health Worker
WHO: World Health Organization
