# Supplementary material for: A systematic review of strategies to increase access to health services among children in low and middle income countries
Source: BMC Health Serv Res. 2017 Apr 5;17:252. doi: 10.1186/s12913-017-2180-9 (PMC5382494; doi:10.1186/s12913-017-2180-9)
Supplement: Additional file 1: — Summary of results of included studies. (DOCX 127 kb) [file 12913_2017_2180_MOESM1_ESM.docx]

**Additional file 1: Summary results of included studies**

**A.** Delivery of services close to home (supply side; non-financial)

|  | **Author (Year)** | **Country and setting** | **Design and population** | **Intervention (Int) and control (Con)** | **Main outcome of interest (HCU= health care utilisation; I=immunisation; C=compliance)** | **Result summary** | **Targeted barrier (according to Peters’ framework)** |
| --- | --- | --- | --- | --- | --- | --- | --- |
| CHWs | | | | | | | |
| 1 | Patouillard (2011)[[1](#_ENREF_1)];  Kweku (2009)[[2](#_ENREF_2)] | Ghana, rural | cRCT; Children aged 3-59 months; n=1,456 | Int: Community based delivery of IPTc by CHW  Cont: Facility based delivery of IPTc | C: Proportion of children who received all for courses of IPTc | Null | Geographic: service location |
| 2 | Seidenburg (2012)[[3](#_ENREF_3)] | Zambia, rural | cRCT; Women aged 14-45 years who had at least one child; n=440 | Int: CHW taught to perform rapid diagnostic tests and administer malaria medication or antibiotics  Con: CHW supplied with malaria medication for treatment of suspected malaria and referred non severe malaria cases to health centre | HCU: First source of care for any illness | Mixed positive | Geographic: service location  Availability: health workers, drugs |
| 3 | Tin (2014)[[4](#_ENREF_4)] | Myanmar, rural | cRCT; Children <5 years; n=104 village tracts | Int: Delivery of ORS and zinc by CHW  Con: Usual services | HCU: Use of ORS plus zinc for diarrhoea | Positive | Geographic: service location  Availability: health workers, drugs |
| 4 | Bojang (1998)[[5](#_ENREF_5)] | The Gambia, rural | cRCT; Children <6 years; n=12,326 | Int: Delivery of IPTc by CHW  Con: Delivery of IPTc by RCH trekking teams | C: coverage of 3 IPTc treatment courses | Positive | Geographic: service location  Availability: health workers, drugs |
| 5 | Brugha (1996)[[6](#_ENREF_6)] | Ghana, rural | cRCT; Children aged 12-18 months; n=419 | Int: Home visits to perform immunisation for children who did not attend appointments  C: No home visits | I: Complete vaccination (BCG, polio, DPT3, measles) | Positive | Geographic: service location  Availability: health workers, drugs |
| Health professional | | | | | | | |
| 7 | Banjeree (2010)[[7](#_ENREF_7)] | India, rural | cRCT; Children 1-3 years, n=2,188 | Int: Well publicised immunisation camps plus/minus food incentive  Con: Usual services | I: Probability of completing the EPI | Positive | Geographic: service location  Availability: demand for services  Acceptability |
| 8 | Simonyan (2013)[[8](#_ENREF_8)] | Mali, urban | CBA; Children aged 0-72 months; n=180 | Int: Home visits for children by health worker who flagged abnormalities with GP and those in need provided with free consultations  Con: Usual care | HCU: Medical consultations for children with reported disease episodes | Positive | Geographic: service location  Financial: cost and prices of services |

##### **B.** Service level improvements (supply side; non-financial)

|  | **Author (Year)** | **Country and setting** | **Design and population** | **Intervention (Int) and control (Con)** | **Main outcome of interest (HCU= health care utilisation; I=immunisation; C=compliance)** | **Result summary** | **Targeted barrier (according to Peters’ framework)** |
| --- | --- | --- | --- | --- | --- | --- | --- |
| Health worker training | | | | | | | |
| 1 | Mohan (2004)[[9](#_ENREF_9)] | India, rural | cRCT; mothers of children < 5 years; n=2,460 | Int: Training for doctors in counselling, communication, clinical skills  Con: Training for doctors in clinical skills alone | HCU: Care seeking behaviour for sick children | Mixed positive | Availability: Health workers  Acceptability: characteristics of health services |
| 2 | Robinson (2001)[[10](#_ENREF_10)] | Indonesia, mixed | CBA; Children age 12-23 months; 12 participating health centres | Int: Immuniser training immuniser peer training programme  Con: No training | I: Number of age appropriate doses DPT1, polio and measles | Positive | Availability: Health workers |
| Scaling up of services | | | | | | | |
| 3 | Ryman (2011)[[11](#_ENREF_11)] | India, rural | CBA; Children 12-23 months, n= 3,681 | Int: Strengthening routine vaccination programme functions  Con: Usual services | I: Proportion children fully vaccinated (BCG, DPT, polio, measles) | Null | Acceptability: characteristics of health services; user's attitudes and expectations.  Geographic: service location.  Availability: health workers, systems improvements. |
| Integration of services | | | | | | | |
| 4 | Dicko (2011)[[12](#_ENREF_12)] | Mali, rural | cRCT; Children aged 0-23 months; n=1,050 | Int: Integration of intermittent preventive treatment for children alongside EPI vaccines  Con: Usual services | I: Proportion of children completely vaccinated (BCG, DPT, polio, measles, yellow fever) | Positive | Acceptability: characteristics of health services  Availability: drugs |
| 5 | McCollum (2012)[[13](#_ENREF_13)] | Malawi, urban | CBA; Children offered HIV testing at immunisation or under 5 clinics; n=1,757 | Int: Integration of early infant diagnosis into immunisation clinics  Con: Early infant diagnosis at under 5 clinic | HCU: Uptake of provider initiated counselling and testing; uptake of PCR testing | Positive | Availability: health workers, drugs, equipment; systems improvements; demand for services |
| 6 | Turan (2015)[[14](#_ENREF_14)]; Washington (2015)[[15](#_ENREF_15)] | Kenya, rural | cRCT; Pregnant HIV positive women >18 years; n=1,172 | Int: Integrated antenatal care, PMTCT and HIV care services  Con: Routine services | HCU: infant HIV testing by 3 and 9 months of age  C: Infant’s ARV use | Mixed negative | Availability: health workers, drugs, equipment; systems improvements; demand for services |
| Combined interventions (primary component service level improvement) | | | | | | | |
| 7 | Arifeen (2009)[[16](#_ENREF_16)] | Bangladesh, rural | cRCT; Families utilising government health facilities; n=20 catchment areas | Int: Health worker training, health systems improvements, family and community activities (eg. Training village health workers)  Con: Usual services | HCU: Proportion of children ill in the last 2 weeks taken to appropriate provider; referral completion  I: Measles vaccination coverage for children aged 12-23 months | Mixed positive | Availability: health workers, systems improvements.  Geographic: service location.  Acceptability: characteristics of health services |
| 8 | Wang (2015)[[17](#_ENREF_17)] | Zambia, rural | cRCT; Children attending under 5 clinic; n=40 facilities | Int: Integration of HIV testing and immunisation services, operational support, training for staff, counselling of caregivers, community awareness campaigns  Con: Usual care | HCU: Average number of DBS tests  I: Average number of DPT1 doses | Null | Availability: health workers; systems improvements; demand for services.  Acceptability: user's attitudes, knowledge and expectations |

##### **C.** Service level improvements (supply side; financial)

|  | **Author (Year)** | **Country and setting** | **Design and population** | **Intervention (Int) and control (Con)** | **Main outcome of interest (HCU= health care utilisation; I=immunisation; C=compliance)** | **Result summary** | **Targeted barrier (according to Peters’ framework)** |
| --- | --- | --- | --- | --- | --- | --- | --- |
| 1 | Schwartz (2004)[[18](#_ENREF_18)] | Cambodia, rural | Non-randomised trial; Children aged 12-23 months; n=1,825 | Int: Contracting in or contracting out health service delivery  Con: Traditional government model | I: Percent children fully immunised (BCG, DPT, polio, measles) | Unclear | Availability: Service level improvements |
| 2 | Basinga (2011)[[19](#_ENREF_19)] | Rwanda, mixed | cRCT; Children < 6 years’ n=166 health facilities | Int: Pay for performance scheme for health workers  Con: Standard service | HCU: Younger than 23 months’ preventive visit; 24-59 months’ preventive visit in previous 4 weeks  I: Aged 12-23 months fully immunised according to national schedule | Mixed positive | Availability: Service level improvements |

##### **D.** Health promotion/**e**ducation (demand side; non-financial)

|  | **Author (Year)** | **Country and setting** | **Design and population** | **Intervention (Int) and control (Con)** | **Main outcome of interest (HCU= health care utilisation; I=immunisation; C=compliance)** | **Result summary** | **Targeted barrier (according to Peters’ framework)** |
| --- | --- | --- | --- | --- | --- | --- | --- |
| Health workers | | | | | | | |
| 1 | Fatugase (2013)[[20](#_ENREF_20)] | Nigeria, rural | Non randomised trial; mothers or caregivers of children < 5 years; n=400 | Int: Structured educational programme on childhood infections for mothers delivered by health worker  Con: No educational programme | HCU: Source of information on infection and treatment; proportion commencing treatment after symptom recognition | Positive | Acceptability: user's attitudes, knowledge and expectations |
| 2 | Usman (2009)[[21](#_ENREF_21)] | Pakistan, urban | RCT; Children visiting EPI centres for DPT1; n=1,506 | Int: Redesigned immunisation card, centre-based education or both  Con: Standard care | I: DPT3 immunisation completed during 90 days followup | Positive | Acceptability: user's attitudes, knowledge and expectations |
| 3 | Usman (2011)[[22](#_ENREF_22)] | Pakistan, rural | RCT; Children visiting EPI centres for DPT1; n=1,500 | Int: Redesigned immunisation card, centre-based education or both  Con: Standard care | I: DPT3 immunisation completed during 90 days followup | Positive | Acceptability: user's attitudes, knowledge and expectations |
| 4 | Bashour (2008)[[23](#_ENREF_23)] | Syria, urban | RCT; women who delivered healthy newborn; n=876 | Int: postpartum home visits by registered midwives to provide information, educate and support women  Con: No home visits | I: Immunisation status at 3 months (according to the national schedule) | Null | Geographic: Location of provider.  Availability: health workers |
| Community health workers | | | | | | | |
| 5 | Bolam (1998)[[24](#_ENREF_24)] | Nepal, urban | RCT; pregnant women; n=540 | Int: Postnatal health education programme for mothers delivered by CHW  Con: No health education | I: Uptake of immunisation at 6 months (DPT, polio, BCG) | Null | Acceptability: user's attitudes, knowledge and expectations |
| 6 | Darmstadt (2010)[[25](#_ENREF_25)] | Bangladesh, rural | cRCT; women aged 15-49 years; n=10,700 pregnancy outcomes | Int: CHW home visits for pregnant women to promote birth and newborn care, refer sick neonates, facilitate compliance  Con: Standard care | HCU: Number of neonates with 1 or more of 10 complications receiving any treatment; or qualified provider | Mixed positive | Geographic: service location  Availability: health workers  Acceptability: user's attitudes, knowledge and expectations |
| 7 | Kirkwood (2013)[[26](#_ENREF_26)] | Ghana, rural | cRCT; pregnancies that ended in livebirth; n=16,329 births | Int: CHW home visits for pregnant women to promote newborn care, assess newborns and refer sick neonates  Con: Standard care | HCU: Care seeking (sick babies taken to hospital or clinic) | Positive | Geographic: service location  Availability: health workers  Acceptability: user's attitudes, knowledge and expectations |
| 8 | Kumar (2008)[[27](#_ENREF_27)] | India, rural | cRCT; pregnant women in the study area; n=3,890 | Int: Package of essential newborn care for pregnant women delivered by CHW  Con: Standard care | HCU: Care seeking providers used for infant; percentage regular clinic visit | Mixed positive | Geographic: service location  Availability: health workers, equipment  Acceptability: user's attitudes, knowledge and expectations |
| 9 | Le Roux (2013) [[28](#_ENREF_28)]; Rotheram-Borus (2014)[[29](#_ENREF_29)] | South Africa, urban | cRCT; pregnant women; n=1,238 | Int: Antenatal and postnatal home visits for pregnant women by CHWs to provide health messages  Con: Standard care | HCU: Infant HIV PCR testing at 6 weeks  I: Number of 6 and 18 month immunisations  C: Infant NVP, AZT post birth | Mixed positive | Availability: health workers  Acceptability: user's attitudes, knowledge and expectations |
| 10 | Owais (2011)[[30](#_ENREF_30)] | Pakistan, urban | cRCT; children < 6 weeks; n=366 | Int: Educational programme for mothers using pictorial cards about vaccinations delivered by CHW  Con: Verbal receipt of health promotional messages delivered by CHWs | I: DPT3/Hep B immunisation rates at 4 months after enrolment | Positive | Acceptability: user's attitudes, knowledge and expectations |
| 11 | Tomlinson (2014)[[31](#_ENREF_31)] | South Africa, urban | cRCT; pregnant women aged 17 and older; n=3,494 | Int: Antenatal and postnatal home visits for pregnant women by CHWs to provide health messages  Con: CHWs provided information on accessing social welfare grants and conducted home visits | HCU: proportion of exposed infants having HIV test at 6 weeks; clinic visit in first week of life; uptake of cotrimoxazole | Mixed positive | Availability: health workers  Acceptability: user's attitudes, knowledge and expectations |
| 12 | Waiswa (2015)[[32](#_ENREF_32)] | Uganda, rural | cRCT; pregnant women and their newborns; n=395 | Int: Antenatal and postnatal home visits for pregnant women by CHWs to provide health messages, assist with birth in absence of skilled care, manage illness where referral not available (sepsis, pneumonia), health facility strengthening  Con: Standard care, facility strengthening | HCU: Care seeking outside home for infants with a danger sign | Null | Geographic: service location  Availability: health workers  Acceptability: user's attitudes, knowledge and expectations |
| Other community member | | | | | | | |
| 13 | Andersson (2009)[[33](#_ENREF_33)] | Pakistan, rural | cRCT; Children < 5 years; n=1,867 | Int: Informed structured discussions with community members on vaccine costs and benefits  Con: No structured discussions | I: Measles vaccination uptake; full DPT vaccination | Mixed positive | Acceptability: user's knowledge, attitudes and expectations |
| 14 | Hanson (2015)[[34](#_ENREF_34)] | Tanzania, rural | cRCT; women of aged 13-49 years; n=1,060 | Int: Home based counselling strategy delivered by female volunteers promoting birth and neonatal care  Con: Standard facility based care | HCU: Referral to hospital for very small babies | Null | Acceptability: user's attitudes, knowledge and expectations  Geographic: service location |
| 15 | Oche (2011)[[35](#_ENREF_35)] | Nigeria, urban | CBA; Children < 2 years; n=358 | Int: Health education about immunisation delivered by community volunteer  Con: Standard care | I: Proportion immunised (DPT3) | Null | Acceptability: user's attitudes, knowledge and expectations |
| Women’s groups | | | | | | | |
| 16 | Fottrell (2013)[[36](#_ENREF_36)] | Bangladesh, rural | cRCT; Women aged 15-49; n=25,321 births | Int: Women’s groups to improve maternal and neonatal health  Con: Standard care | HCU: Infant received check up in the first 6 weeks by formal provider | Null | Acceptability: user's attitudes, knowledge and expectations |
| 17 | Houweling (2013);[[37](#_ENREF_37)] Tripathy (2010)[[38](#_ENREF_38)] | India, rural | cRCT; Women aged 15-49 years; n=18,775 births | Int: Women’s groups to improve maternal and neonatal health  Con: Standard care | HCU: Care seeking behaviour in the event of infant illness; post-natal check-up for baby at medical facility | Null | Acceptability: user's attitudes, knowledge and expectations  Availability: system improvements; demand for services |
| 18 | Manandhar (2004)[[39](#_ENREF_39)] | Nepal, rural | cRCT; Women aged 15-49 years; n=6,275 births | Int: Women’s groups to improve maternal and neonatal health, strengthening of health services, training of healthcare workers, CHWs, and TBAs  Con: Standard care | HCU: Proportion of infants taken to health facility in event of illness | Positive | Acceptability: user's attitudes, knowledge and expectations  Availability: system improvements |
| 19 | More (2012)[[40](#_ENREF_40)] | India, rural | cRCT; Women who joined groups; n=18,197 births | Int: Women’s groups to improve maternal and neonatal health  Con: No women’s groups | HCU: Clinic care for specified newborn illness within the first 24 hours  I: Infant BCG vaccine | Null | Acceptability: user's attitudes, knowledge and expectations |
| Combined interventions (primary component education) | | | | | | | |
| 20 | Azad (2010)[[41](#_ENREF_41)] | Bangladesh, rural | cRCT; Women aged 15-49 years; n=30,952 births | Int: Women’s groups to improve maternal and neonatal health outcomes, health services strengthening  Con: No women’s groups, health services strengthening | HCU: Health care seeking behaviour in the event of an illness | Null | Acceptability: user's attitudes, knowledge and expectations  Availability: system improvements; demand for services |
| 21 | Bari (2006)[[42](#_ENREF_42)] | Bangladesh, rural | cRCT; Infants and caregivers; n=4,343 | Int: Health education of families, identification of sick newborns in the community by CHW, health systems strengthening and strengthening of referral systems (including provision of free care and referrals)  Int: Usual services | HCU: Care seeking from qualified providers; care seeking from hospital | Positive | Geographic: service location  Acceptability: user's knowledge, attitudes and perceptions  Financial: cost and prices of services |
| 22 | Brenner (2011)[[43](#_ENREF_43)] | Uganda, rural | CBA; Children < 5 years; n=1,118 | Int: Health promotion for children delivered by CHW, illness management, community development  Con: Usual services | I: Measles vaccination coverage | Positive | Geographic: service location  Availability: health workers  Acceptability: user's attitudes, knowledge and expectations |
| 23 | Mazumder (2014)[[44](#_ENREF_44)] | India, mixed | cRCT; infants; n=29,667 births | Int: Home visits by CHWs, training in improved case management of sick children, women’s groups, strengthening of health systems  Con: Standard care | HCU: Clinic care for severe newborn illness within the first 24 hours  I: Infant BCG vaccine | Null | Geographic: service location  Availability: systems improvements  Acceptability: user's attitudes and expectations |

##### **E.** Text messages (demand side; non-financial)

|  | **Author (Year)** | **Country and setting** | **Design and population** | **Intervention (Int) and control (Con)** | **Main outcome of interest (HCU= health care utilisation; I=immunisation; C=compliance)** | **Result summary** | **Targeted barrier (according to Peters’ framework)** |
| --- | --- | --- | --- | --- | --- | --- | --- |
| 1 | Bangure (2015)[[45](#_ENREF_45)] | Zimbabwe, mixed | RCT; mother or caregiver; n=304 | Int: SMS vaccine appointment reminders and health education at 6, 10 and 14 weeks of age  Con: Routine health education only | I: Receipt of scheduled vaccines at 6,10, 14 weeks | Positive | Acceptability: user's attitudes, knowledge and expectations |
| 2 | Bigna (2014)[[46](#_ENREF_46)] | Cameroon, mixed | RCT; caregivers of children who are infected/exposed to HIV; n=242 | Int: HIV appointment reminders by i) SMS ii) Phone call iii) Both  Con: No reminder | HCU: Proportion of patients attending the previously scheduled appointment | Positive | Acceptability: user's attitudes, knowledge and expectations |
| 3 | Finocchano-Kessler (2014)[[47](#_ENREF_47)] | Kenya, urban | Historically controlled study; mother infant pairs presenting to EID; n=843 | Int: Early infant diagnosis for HIV SMS reminders to mothers for results, treatment, routine testing due  Con: Historical controls | HCU: Retention in EID care at 9 months | Positive | Acceptability: user's attitudes, knowledge and expectations |
| 4 | Odeny (2014)[[48](#_ENREF_48)] | Kenya, urban | RCT; HIV positive women attending ANC; n=388 | Int: SMS health promotional messages during pregnancy for HIV positive women | HCU: Infant HIV testing uptake | Positive | Acceptability: user's attitudes, knowledge and expectations |
| 5 | Schlumberger (2015)[[49](#_ENREF_49)] | Burkina Faso, urban | RCT; mothers attending first EPI appointment; n=521 mothers | Int: SMS appointment reminders for EPI vaccinations  Con: Standard care | I: Uptake of vaccinations (BCG, DPT, HiB, Hep B, pneumococcal, antirotovirus, polio, rubella, yellow fever) | Mixed positive | Geographic: service location  Availability: health workers  Acceptability: user's attitudes, knowledge and expectations |

##### **F.** Financial or other incentive (demand side; financial)

|  | **Author (Year)** | **Country and setting** | **Design and population** | **Intervention (Int) and control (Con)** | **Main outcome of interest (HCU= health care utilisation; I=immunisation; C=compliance)** | **Result summary** | **Targeted barrier (according to Peters’ framework)** |
| --- | --- | --- | --- | --- | --- | --- | --- |
| Cash transfers | | | | | | | |
| 1 | Akresh (2012)[[50](#_ENREF_50)] | Burkina Faso, rural | cRCT; Children<15 years; n=2,559 | Int: Conditional or unconditional cash transfers made to mother or father  Con: No cash transfer | HCU: Routine preventative health clinic visits | Mixed positive | Financial: recipient resources; willingness to pay |
| 2 | Beck (2015)[[51](#_ENREF_51)] | India, rural | cRCT; Villages; n=2,034 households per village | Int: Unconditional cash transfer  Con: No cash transfer | I: Proportion children fully vaccinated (BCG, polio, DPT, MMR) | Null | Financial: recipient resources; willingness to pay |
| 3 | Barham (2009)[[52](#_ENREF_52)] | Nicaragua, rural | cRCT; Children aged 0-35 months; n=2,229 | Int: Conditional cash transfer to mother (*Red de Proteccion Social*)  Con: No cash transfer | I: Proportion children fully vaccinated for all 4 vaccines (BCG, measles, polio, DPT) | Null | Financial: recipient resources; willingness to pay |
| 4 | Robertson (2013)[[53](#_ENREF_53)] | Zimbabwe, mixed | cRCT; Children<18 years; n=2,507 | Int: Conditional or unconditional cash transfers  Con: No cash transfer | I: Proportion of children with up to date vaccinations (measles, BCG, polio, DPT) | Null | Financial: recipient resources; willingness to pay |
| 5 | Macours (2012)[[54](#_ENREF_54)] | Nicuragua, rural | cRCT; poor households; n=4,021 households | Int: Conditional cash transfer (Atencion a Crisis)  Con: No transfer | HCU: Use of preventive health services | Mixed positive | Financial: recipient resources; willingness to pay |
| Fee exemptions | | | | | | | |
| 6 | Abdu (2004)[[55](#_ENREF_55)] | Sudan, urban and rural | cRCT; Children<3 years and pregnant women; n=8 health centres | Int: Health centre user fee exemptions  Con: No exemption | HCU: Number of children with malaria seen at health centre | Positive | Financial: cost of services |
| 7 | Ansah (2009)[[56](#_ENREF_56)]; Ansah (2013)[[57](#_ENREF_57)]; Powell- Jackson (2013)[[58](#_ENREF_58)] | Ghana, rural | cRCT; Children aged 6-59 months; n=4,765 | Int: Removal of user fees  Con: Paid user fees | HCU: Number of clinic visits per year | Mixed positive | Financial: cost of services |
| Incentive schemes | | | | | | | |
| 8 | Chandir (2010)[[59](#_ENREF_59)] | Pakistan, urban | CBA; Infants 0-6 months; n=4,545 | Int: Food/medicine incentive at each immunisation visit  Con: No incentive | I: DPT3 immunisation at 18 weeks | Positive | Financial: Recipient resources and willingness to pay |
| 9 | Kundu (2012)[[60](#_ENREF_60)] | India, urban | Historically controlled study; Children 2-12 years, n=180 | Int: Provision of supplementary nutrition for children attending HIV/AIDS clinic  Con: No supplementary nutrition | HCU: Percentage regular clinic visits | Positive | Financial: Recipient resources and willingness to pay |
| Combined interventions (primary component financial) | | | | | | | |
| 10 | Ridde (2013)[[61](#_ENREF_61)] | Burkina Faso, rural | ITS, Children <5 years; n=112,724 observations | Int: Fee exemption for curative care, health education, strengthening of services  Con: No fee exemption, standard care | HCU: Health centre utilisation | Positive | Financial: cost of services  Availability: strengthening of services |
| 11 | Galasso (2011)[[62](#_ENREF_62)] | Chile, rural | CBA; poor households; n=12,900 households | Int: Conditional cash transfer, strengthening of services (*Chile Solidario*)  Con: No cash transfer, standard services | HCU: Children under 6 with regular check ups | Null | Financial: recipient resources; willingness to pay  Availability: strengthening of services |
| 12 | Morris (2004)[[63](#_ENREF_63)] | Honduras, rural | cRCT; Children <3 years and pregnant women, n=70 municipalities | Int: Conditional cash transfer, strengthening of services (*Programe de asignacion familiar)*  Con: No cash transfer, standard services | HCU: Proportion of children take to health centre in last 30 days  I: Measles, DPT1 coverage | Mixed positive | Financial: recipient resources; willingness to pay  Availability: strengthening of services |

**References for tables**

1. Patouillard, E., L. Conteh, J. Webster, M. Kweku, D. Chandramohan, and B. Greenwood, *Coverage, adherence and costs of intermittent preventive treatment of malaria in children employing different delivery strategies in Jasikan, Ghana.* PLoS ONE, 2011. **24**.

2. Kweku, M., J. Webster, M. Adjuik, S. Abudey, B. Greenwood, and D. Chandramohan, *Options for the delivery of intermittent preventive treatment for malaria to children: a community randomised trial.* PLoS One, 2009. **4**(9): p. e7256.

3. Seidenberg, P.D., D.H. Hamer, H. Iyer, P. Pilingana, K. Siazeele, B. Hamainza, et al., *Impact of integrated community case management on health-seeking behavior in rural Zambia.* Am J Trop Med Hyg, 2012. **87**(5 Suppl): p. 105-10.

4. Tin, A., D. Montagu, K. Hnin Su Su, W. Zaw, S. Ang Kyaw, and W. McFarland, *Impact of a social franchising program on uptake of oral rehydration solution plus zinc for childhood diarrhea in Myanmar: a community-level randomized controlled trial.* Journal of Tropical Pediatrics, 2014. **60**(3): p. 189-197.

5. Bojang, K.A., F. Akor, L. Conteh, E. Webb, O. Bittaye, D.J. Conway, et al., *Two strategies for the delivery of IPTc in an area of seasonal malaria transmission in The Gambia: a randomised controlled trial.* PLoS Medicine, 2011. **8**(2).

6. Brugha, R.F. and J.P. Kevany, *Maximizing immunization coverage through home visits: a controlled trial in an urban area of Ghana.* Bull World Health Organ, 1996. **74**(5): p. 517-24.

7. Banerjee, A.V., E. Duflo, R. Glennerster, and D. Kothari, *Improving immunisation coverage in rural India: clustered randomised controlled evaluation of immunisation campaigns with and without incentives.* Bmj, 2010. **340**(c2220).

8. Simonyan, D., M.P. Gagnon, T. Duchesne, and A. Roos-Weil, *Effects of a telehealth programme using mobile data transmission on primary healthcare utilisation among children in Bamako, Mali.* J Telemed Telecare, 2013. **19**(6): p. 302-6.

9. Mohan, P., S.D. Iyengar, J. Martines, S. Cousens, and K. Sen, *Impact of counselling on careseeking behaviour in families with sick children: cluster randomised trial in rural India.* BMJ, 2004. **329**(7460): p. 266.

10. Robinson, J.S., B.R. Burkhalter, B. Rasmussen, and R. Sugiono, *Low-cost on-the-job peer training of nurses improved immunization coverage in Indonesia.* Bull World Health Organ, 2001. **79**(2): p. 150-8.

11. Ryman, T.K., A. Trakroo, A. Wallace, S.K. Gupta, K. Wilkins, P. Mehta, et al., *Implementation and evaluation of the Reaching Every District (RED) strategy in Assam, India, 2005-2008.* Vaccine, 2011. **29**(14): p. 2555-60.

12. Dicko, A., S.O. Toure, M. Traore, I. Sagara, O.B. Toure, M.S. Sissoko, et al., *Increase in EPI vaccines coverage after implementation of intermittent preventive treatment of malaria in infant with Sulfadoxine -pyrimethamine in the district of Kolokani, Mali: results from a cluster randomized control trial.* BMC Public Health, 2011. **11**: p. 573.

13. McCollum, E.D., D.C. Johnson, C.S. Chasela, L.D. Siwande, P.N. Kazembe, D. Olson, et al., *Superior uptake and outcomes of early infant diagnosis of HIV services at an immunization clinic versus an "under-five" general pediatric clinic in Malawi.* JAIDS, Journal of Acquired Immune Deficiency Syndromes, 2012. **60**(4): p. e107-e110.

14. Turan, J.M., M. Onono, R.L. Steinfeld, S.B. Shade, K. Owuor, S. Washington, et al., *Implementation and operational research: effects of antenatal care and HIV treatment integration on elements of the PMTCT cascade: results from the SHAIP cluster-randomized controlled trial in Kenya.* JAIDS, Journal of Acquired Immune Deficiency Syndromes, 2015. **69**(5): p. e172-e181.

15. Washington, S., K. Owuor, J.M. Turan, R.L. Steinfeld, M. Onono, S.B. Shade, et al., *Implementation and operational research: effect of integration of HIV care and treatment into antenatal care clinics on mother-to-child HIV transmission and maternal outcomes in Nyanza, Kenya: results from the SHAIP cluster randomized controlled trial.* JAIDS, Journal of Acquired Immune Deficiency Syndromes, 2015. **69**(5): p. e164-e171.

16. Arifeen, S.E., D.M. Hoque, T. Akter, M. Rahman, M.E. Hoque, K. Begum, et al., *Effect of the Integrated Management of Childhood Illness strategy on childhood mortality and nutrition in a rural area in Bangladesh: a cluster randomised trial.* Lancet, 2009. **374**(9687): p. 393-403.

17. Wang, P.C., A. Mwango, S. Moberley, B.J. Brockman, A.L. Connor, P. Kalesha-Masumbu, et al., *A cluster randomised trial on the impact of integrating early infant HIV diagnosis with the expanded programme on immunization on immunization and HIV testing rates in rural health facilities in Southern Zambia.* PLoS ONE, 2015. **10**(10).

18. Schwartz, J.B. and I. Bhushan, *Improving immunization equity through a public-private partnership in Cambodia.* Bull World Health Organ, 2004. **82**(9): p. 661-7.

19. Basinga, P., P.J. Gertler, A. Binagwaho, A.L.B. Soucat, J. Sturdy, and C.M.J. Vermeersch, *Effect on maternal and child health services in Rwanda of payment to primary health-care providers for performance: an impact evaluation.* Lancet, 2011. **377**(9775): p. 1421-1428.

20. Fatugase, O.M., O.E. Amoran, and O.K. Fatugase, *The impact of health education intervention on perception and treatment seeking behaviour about childhood infections among caregivers in rural communities in western Nigeria.* British Journal of Medicine and Medical Research, 2013. **3**(4): p. 1331-1343.

21. Usman, H.R., S. Akhtar, F. Habib, and I. Jehan, *Redesigned immunization card and center-based education to reduce childhood immunization dropouts in urban Pakistan: a randomized controlled trial.* Vaccine, 2009. **27**(3): p. 467-72.

22. Usman, H.R., M.H. Rahbar, S. Kristensen, S.H. Vermund, R.S. Kirby, F. Habib, et al., *Randomized controlled trial to improve childhood immunization adherence in rural Pakistan: redesigned immunization card and maternal education.* Trop Med Int Health, 2011. **16**(3): p. 334-42.

23. Bashour, H.N., M.H. Kharouf, A.A. Abdulsalam, K. El Asmar, M.A. Tabbaa, and S.A. Cheikha, *Effect of postnatal home visits on maternal/infant outcomes in Syria: a randomized controlled trial.* Public Health Nurs, 2008. **25**(2): p. 115-25.

24. Bolam, A., D.S. Manandhar, P. Shrestha, M. Ellis, and A.M. Costello, *The effects of postnatal health education for mothers on infant care and family planning practices in Nepal: a randomised controlled trial.* Bmj, 1998. **316**(7134): p. 805-11.

25. Darmstadt, G.L., Y.J. Choi, S.E. Arifeen, B. Sanwarul, S.M. Rahman, I. Mannan, et al., *Evaluation of a cluster-randomized controlled trial of a package of community-based maternal and newborn interventions in Mirzapur, Bangladesh.* PLoS ONE, 2010. **59**.

26. Kirkwood, B.R., A. Manu, A.H.t. Asbroek, S. Soremekun, B. Weobong, T. Gyan, et al., *Effect of the Newhints home-visits intervention on neonatal mortality rate and care practices in Ghana: a cluster randomised controlled trial.* Lancet, 2013. **381**(9884): p. 2184-2192.

27. Kumar, V., S. Mohanty, A. Kumar, R.P. Misra, M. Santosham, S. Awasthi, et al., *Effect of community-based behaviour change management on neonatal mortality in Shivgarh, Uttar Pradesh, India: a cluster-randomised controlled trial.* The Lancet, 2008. **372**(9644): p. 1151-1162.

28. le Roux, I.M., M. Tomlinson, J.M. Harwood, M.J. O'Connor, C.M. Worthman, N. Mbewu, et al., *Outcomes of home visits for pregnant mothers and their infants: a cluster randomized controlled trial.* AIDS, 2013. **27**(9): p. 1461-71.

29. Rotheram-Borus, M.J., M. Tomlinson, I.M. le Roux, J.M. Harwood, S. Comulada, M.J. O'Connor, et al., *A cluster randomised controlled effectiveness trial evaluating perinatal home visiting among South African mothers/infants.* PLoS One, 2014. **9**(10): p. e105934.

30. Owais, A., B. Hanif, A.R. Siddiqui, A. Agha, and A.K. Zaidi, *Does improving maternal knowledge of vaccines impact infant immunization rates? A community-based randomized-controlled trial in Karachi, Pakistan.* BMC Public Health, 2011. **11**: p. 239.

31. Tomlinson, M., T. Doherty, P. Ijumba, D. Jackson, J. Lawn, L.A. Persson, et al., *Goodstart: a cluster randomised effectiveness trial of an integrated, community-based package for maternal and newborn care, with prevention of mother-to-child transmission of HIV in a South African township.* Tropical Medicine and International Health, 2014. **19**(3): p. 256-266.

32. Waiswa, P., G. Pariyo, K. Kallander, J. Akuze, G. Namazzi, E. Ekirapa-Kiracho, et al., *Effect of the Uganda Newborn Study on care-seeking and care practices: a cluster-randomised controlled trial.* Glob Health Action, 2015. **8**: p. 24584.

33. Andersson, N., A. Cockcroft, N.M. Ansari, K. Omer, M. Baloch, A. Ho Foster, et al., *Evidence-based discussion increases childhood vaccination uptake: a randomised cluster controlled trial of knowledge translation in Pakistan.* BMC Int Health Hum Rights, 2009. **9 Suppl 1**: p. S8.

34. Hanson, C., F. Manzi, E. Mkumbo, K. Shirima, S. Penfold, Z. Hill, et al., *Effectiveness of a home-based counselling strategy on neonatal care and survival: a cluster-randomised trial in six districts of rural southern Tanzania.* PLoS Medicine, 2015. **12**(9).

35. Oche, M.O., A.S. Umar, M.T.O. Ibrahim, and K. Sabitu, *An assessment of the impact of health education on maternal knowledge and practice of childhood immunization in Kware, Sokoto State.* Journal of Public Health and Epidemiology, 2011. **3**(10): p. 440-447.

36. Fottrell, E., K. Azad, A. Kuddus, L. Younes, S. Shaha, T. Nahar, et al., *The effect of increased coverage of participatory women's groups on neonatal mortality in Bangladesh: A cluster randomized trial.* JAMA Pediatr, 2013. **167**(9): p. 816-25.

37. Houweling, T.A., P. Tripathy, N. Nair, S. Rath, S. Rath, R. Gope, et al., *The equity impact of participatory women's groups to reduce neonatal mortality in India: secondary analysis of a cluster-randomised trial.* Int J Epidemiol, 2013. **42**(2): p. 520-32.

38. Arora, M., M. Stigler, V. Gupta, S. Bassi, P. Dhavan, V. Tripathy, et al., Circulation. Conference: World Congress of Cardiology Scientific Sessions, 2010. **122**(2).

39. Manandhar, D.S., D. Osrin, B.P. Shrestha, N. Mesko, J. Morrison, K.M. Tumbahangphe, et al., *Effect of a participatory intervention with women's groups on birth outcomes in Nepal: cluster-randomised controlled trial.* The Lancet, 2004. **364**(9438): p. 970-979.

40. More, N.S., U. Bapat, S. Das, G. Alcock, S. Patil, M. Porel, et al., *Community mobilization in Mumbai slums to improve perinatal care and outcomes: a cluster randomized controlled trial.* PLoS Med, 2012. **9**(7): p. e1001257.

41. Azad, K., S. Barnett, B. Banerjee, S. Shaha, K. Khan, A.R. Rego, et al., *Effect of scaling up women's groups on birth outcomes in three rural districts in Bangladesh: a cluster-randomised controlled trial.* Lancet, 2010. **375**(9721): p. 1193-202.

42. Bari, S., I. Mannan, M.A. Rahman, G.L. Darmstadt, M.H.R. Seraji, A.H. Baqui, et al., *Trends in Use of Referral Hospital Services for Care of Sick Newborns in a Community-based Intervention in Tangail District, Bangladesh.* J Health Popul Nutr, 2006. **24**.

43. Brenner, J.L., J. Kabakyenga, T. Kyomuhangi, K.A. Wotton, C. Pim, M. Ntaro, et al., *Can volunteer community health workers decrease child morbidity and mortality in southwestern Uganda? An impact evaluation.* PLoS One, 2011. **6**(12): p. e27997.

44. Mazumder, S., S. Taneja, R. Bahl, P. Mohan, T.A. Strand, H. Sommerfelt, et al., *Effect of implementation of integrated management of neonatal and childhood illness programme on treatment seeking practices for morbidities in infants: cluster randomised trial.* BMJ, 2014. **349**: p. g4988.

45. Bangure, D., D. Chirundu, N. Gombe, T. Marufu, G. Mandozana, M. Tshimanga, et al., *Effectiveness of short message services reminder on childhood immunization programme in Kadoma, Zimbabwe - a randomized controlled trial, 2013.* BMC Public Health, 2015. **15**(137).

46. Bigna, J.J.R., J.J.N. Noubiap, C. Kouanfack, C.S. Plottel, and S. Koulla-Shiro, *Effect of mobile phone reminders on follow-up medical care of children exposed to or infected with HIV in Cameroon (MORE CARE): a multicentre, single-blind, factorial, randomised controlled trial.* The Lancet Infectious Diseases, 2014. **14**(7): p. 600-608.

47. Finocchario-Kessler, S., B.J. Gautney, S. Khamadi, V. Okoth, K. Goggin, J.K. Spinler, et al., *If you text them, they will come: using the HIV infant tracking system to improve early infant diagnosis quality and retention in Kenya. (Special Issue: Children born into families affected by HIV.).* Aids, 2014. **28**(Suppl. 3): p. S313-S321.

48. Odeny, T.A., E.A. Bukusi, C.R. Cohen, K. Yuhas, C.S. Camlin, and R.S. McClelland, *Texting improves testing: a randomized trial of two-way SMS to increase postpartum prevention of mother-to-child transmission retention and infant HIV testing.* AIDS, 2014. **28**(15): p. 2307-12.

49. Schlumberger, M., A. Bamoko, T.M. Yameogo, F. Rouvet, R. Ouedraogo, B. Traore, et al., *Positive impact on the expanded program on immunization when sending call-back SMS through a Computerized Immunization Register, Bobo Dioulasso (Burkina Faso).* Bulletin de la Societe de Pathologie Exotique, 2015. **108**(5): p. 349-354.

50. Akresh, R., D.d. Walque, and H. Kazianga, *Alternative cash transfer delivery mechanisms: impacts on routine preventative health clinic visits in Burkina Faso.* IZA Discussion Papers Forschungsinstitut zur Zukunft der Arbeit, 2012. **28**(25).

51. Beck, S., A.M. Pulkki-Brannstrom, and M. San Sebastian, *Basic income - healthy outcome? Effects on health of an Indian basic income pilot project: a cluster randomised trial.* Journal of Development Effectiveness, 2015. **7**(1): p. 111-126.

52. Barham, T. and J.A. Maluccio, *Eradicating diseases: The effect of conditional cash transfers on vaccination coverage in rural Nicaragua.* J Health Econ, 2009. **28**(3): p. 611-21.

53. Robertson, L., P. Mushati, J.W. Eaton, L. Dumba, G. Mavise, J. Makoni, et al., *Effects of unconditional and conditional cash transfers on child health and development in Zimbabwe: a cluster-randomised trial.* Lancet, 2013. **381**(9874): p. 1283-1292.

54. Macours, K., N. Schady, and R. Vakis, *Cash Transfers, Behavioral Changes, and Cognitive Development in Early Childhood: Evidence from a Randomized Experiment.* American Economic Journal: Applied Economics, 2012. **4**(2): p. 247-273.

55. Abdu, Z., Z. Mohammed, I. Bashier, and B. Eriksson, *The impact of user fee exemption on service utilization and treatment seeking behaviour: the case of malaria in Sudan.* Int J Health Plann Manage, 2004. **19 Suppl 1**: p. S95-106.

56. Ansah, E.K., S. Narh-Bana, S. Asiamah, V. Dzordzordzi, K. Biantey, K. Dickson, et al., *Effect of removing direct payment for health care on utilisation and health outcomes in Ghanaian children: a randomised controlled trial.* PLoS Med, 2009. **6**(1): p. e1000007.

57. Ansah, E.K. and T. Powell-Jackson, *Can we trust measures of healthcare utilization from household surveys?* BMC Public Health, 2013. **13**: p. 853.

58. Powell-Jackson, T., K. Hanson, C.J.M. Whitty, and E.K. Ansah, *Who benefits from free healthcare? Evidence from a randomized experiment in Ghana.* Journal of Development Economics, 2014. **107**: p. 305-319.

59. Chandir, S., A.J. Khan, H. Hussain, H.R. Usman, S. Khowaja, N.A. Halsey, et al., *Effect of food coupon incentives on timely completion of DTP immunization series in children from a low-income area in Karachi, Pakistan: a longitudinal intervention study.* Vaccine, 2010. **28**(19): p. 3473-8.

60. Kundu, C.K., S. Moumita, S. Mihir, B. Subhasish, and C. Sukanta, *Food supplementation as an incentive to improve pre-antiretroviral therapy clinic adherence in HIV-positive children - experience from eastern India.* Journal of Tropical Pediatrics, 2012. **58**(1): p. 31-37.

61. Ridde, V., S. Haddad, and R. Heinmuller, *Improving equity by removing healthcare fees for children in Burkina Faso.* Journal of Epidemiology & Community Health, 2013. **67**(9): p. 751-757.

62. Galasso, E., *Alleviating Extreme Poverty in Chile: The Short Term Effects of Chile Solidario.* Estudios de Economía, 2011. **38**(1): p. 101.

63. Morris, S.S., R. Flores, P. Olinto, and J.M. Medina, *Monetary incentives in primary health care and effects on use and coverage of preventive health care interventions in rural Honduras: cluster randomised trial.* The Lancet, 2004. **364**(9450): p. 2030-2037.
